# Cardiac MRI characteristics in patients with borderline pulmonary hypertension: results from the ASPIRE registry

**DOI:** 10.1186/1532-429X-17-S1-P350

**Published:** 2015-02-03

**Authors:** Henry Walker, Jim M Wild, Nehal Hussain, Charlie Elliot, Robin Condliffe, David G Kiely, Andrew J Swift

**Affiliations:** 1 Department of cardiovascular Science, University of Sheffield, Sheffield, UK; 2 Sheffield Pulmonary Vascular Disease Unit, Sheffield Teaching Hospitals NHS Trust, Sheffield, UK

## Background

Pulmonary hypertension (PH) is a severe disorder defined as a mean pulmonary artery pressure (mPAP) ≥ 25mmHg at right heart catheterization. Previous research indicates the mPAP of healthy volunteers is 14mmHg (±3.3mmHg) at rest, and patients with mPAP of 21 to 24mmHg are considered as borderline PH.

This study investigates demographic, co-morbidities, invasive haemodynamics and cardiac magnetic resonance (CMR) imaging characteristics of patients with borderline PH.

## Methods

Consecutive patients who underwent CMR at 1.5T at a large volume pulmonary hypertension centre were identified. CMR imaging was performed on a whole body scanner GE HDx at 1.5T (GE Healthcare, Milwaukee, USA) with an 8 channel cardiac coil. Exclusion criteria were non-diagnostic imaging or right heart catheterisation (RHC) >24 hours from time of CMR. Biventricular volume, mass and function, pulmonary artery form and function, left atrial (LA) volume and inter-ventricular septal (IVS) angle were assessed. All demographic, invasive haemodynamic and CMR and haemodynamic variables were compared between patients with normal pulmonary artery pressure (mPAP ≤ 20mmHg) and a borderline group (20 ≤ mPAP ≤ 24 mmHg) using an independent samples t-test, and bilogistic regression.

## Results

797 consecutive patients with suspected PH referred for CMR between April 2012 and March 2014 were identified. 83 patients had mPAP of less than 25, and 72 were included in this study having CMR within 24 hours of RHC. There was no significant difference between the age, sex distribution, body surface area, heart rate or cardiac output of the two groups. MRAP, PCWP and as expected PVR were significantly elevated in the borderline PH group. RVEDVI (p=0.025), IVS angle in systole (p=0.024) and LA volume (p=0.047) are all significantly higher in patients with borderline PH than those with mPAP<20mmHg. RV functional measurements were not elevated in patients with borderline PH. At bilogistic regression borderline PH was independently associated with elevated IVS angle. MPAP correlated significantly with RVEDVI (p=0.040) and IVS angle (p=0.046), and PVR correlated strongly with PA relative area change (p=0.001).

## Conclusions

Patients with borderline PH have higher indexed RV end-diastolic volume and greater septal deviation but preserved RV functional metrics when compared to patients with normal pulmonary artery pressure. Elevated PCWP and LA volume likely relates to the higher proportion of patients with underlying left heart disease in the borderline PH group. This is the first study to demonstrate changes in CMR cardiac morphology in patients with borderline pulmonary hypertension, these findings challenge the current definition of PH.

## Funding

AJS and JMW have received funding from the National Institute for Health Research (NIHR). JMW is also funded by the Engineering and Physical Sciences Research Council (EPSRC). DC receives funding from Bayer Schering.

**Figure 1:**
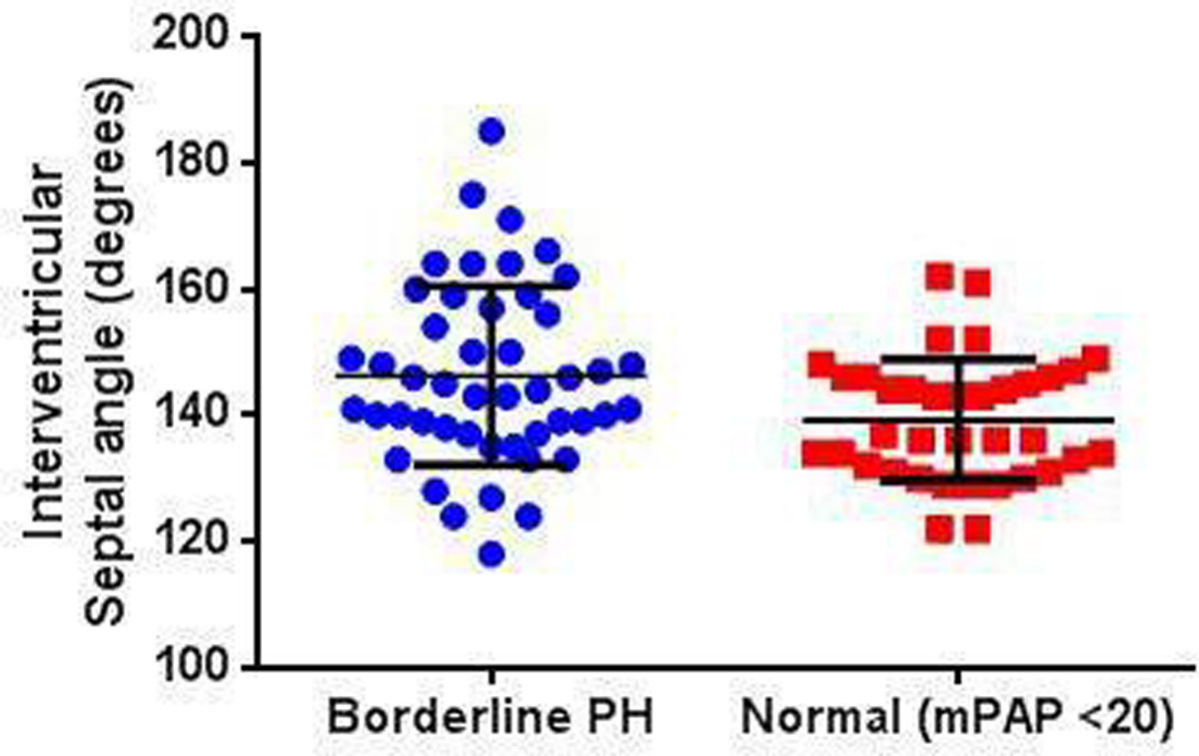
Dot plot illustrating higher mean interventricular septal angle in patients with Borderline PH (mPAP 21-24) compared to patients with normal pulmonary artery pressure (mPAP ≤ 20mmHg).

